# Biochemical Changes in Irradiated Oral Mucosa: A FTIR Spectroscopic Study

**DOI:** 10.3390/bios9010012

**Published:** 2019-01-13

**Authors:** Helena Ukkonen, Simo Vuokila, Jopi J. W. Mikkonen, Hannah Dekker, Engelbert A. J. M. Schulten, Elisabeth Bloemena, Arto Koistinen, Tulio A. Valdez, Arja M. Kullaa, Surya Pratap Singh

**Affiliations:** 1 Research Group of Oral Health Sciences, Faculty of Medicine, University of Oulu, and Oulu University Hospital, P.O. Box 8000, 90014 Oulu, Finland; Helena.Ukkonen@oulu.fi (H.U.); arja.kullaa@uef.fi (A.M.K.); 2 Medical Research Center Oulu, Oulu University Hospital, P.O. Box 5000, 90029 Oulu, Finland; 3 SIB Labs, University of Eastern Finland, P.O. Box 1627, 70211 Kuopio, Finland; simovuo@student.uef.fi (S.V.); jopi.mikkonen@uef.fi (J.J.W.M.); arto.koistinen@uef.fi (A.K.); 4 Institute of Dentistry, University of Eastern Finland, Kuopio campus, and Educational Dental Clinic, Kuopio University Hospital, P.O. Box 1627, 70211 Kuopio, Finland; 5 Amsterdam UMC and Academic Centre for Dentistry Amsterdam (ACTA), Vrije Universiteit Amsterdam, Department of Oral and Maxillofacial Surgery/Oral Pathology, De Boelelaan 1117, 1081 HV Amsterdam, The Netherlands; ha.dekker@vumc.nl (H.D.); eajm.schulten@vumc.nl (E.A.J.M.S.); e.bloemena@vumc.nl (E.B.); 6 Department of Otolaryngology, School of Medicine, Stanford University, Patlo Alto, CA 94305, USA; tvaldez1@stanford.edu

**Keywords:** FTIR spectroscopy, head and neck cancer, radiotherapy

## Abstract

Radiation exposure during the course of treatment in head and neck cancer (HNC) patients can induce both structural and biochemical anomalies. The present study is focused on utilizing infrared imaging for the identification of the minor biochemical alterations in the oral mucosa. Chemical maps generated using glycoprotein band indicates its differential distribution along the superficial layer. Spectra extracted from this layer suggests changes in overall nucleic acid and protein content in response to the therapeutic irradiation. Discrimination among control and irradiated groups have been achieved using principal component analysis. Findings of this preliminary study further support prospective utilization of Fourier Transform InfraRed (FTIR) imaging as a non-destructive, label-free tool for objective assessment of the oral mucosa in patient groups with or without radiation therapy.

## 1. Introduction

Radiation therapy (RT) in combination with chemotherapy and surgical resection of pathological tissue is common treatment for head and neck cancers [1]. Even though there is significant advancement in the treatment and diagnosis of cancers, mortality and morbidity rates remain high [2]. The common short-term (acute) complication of RT is the inflammation and ulceration of oral mucosa, called oral mucositis [3]. Long-term (chronic) adverse effects include tissue atrophy, xerostomia and hyposalivation, radiation induced fibrosis, and osteoradionecrosis (ORN). Radiation affects the malignant cells as well as the surrounding healthy tissue, and radiation-induced fibrosis is the cause of damage due to irradiation to the soft tissue [4,5].

Normal oral mucosa is defended and moisturized by salivary and glycoprotein complexes called mucosal pellicle, with loosely attached salivary film [6]. Superficial cells of the buccal mucosa express transmembrane glycoprotein Mucin 1 (MUC 1) and are covered by many “wrinkles”, or folds called microplicae (MPL), on the cell surface. It provides the underlying foundation for the stable mucosal pellicle [7,8]. This complex forms a protective barrier against pathogens. Previous studies have successfully implied that the normal MPL structure is disrupted in different oral diseases [9,10,11]. Irradiation-induced changes in MPL structures in terms of uneven cell boundaries and total destruction of cell membranes had been demonstrated by electron microscopy in patient samples with diagnosed ORN [12].

FTIR spectroscopy is an absorption based optical technique. Major benefits of using FTIR imaging over traditional methods is that it is non-disruptive, label-free, and requires very limited amount of specimen. It provides a biochemical fingerprint consisting of information about the content, structure, and chemical modification of major biomolecules of the investigated sample [13]. In case of biological samples, a snapshot of modification in DNA, RNA, proteins, and carbohydrates induced by external or interventions can be objectively elucidated with FTIR imaging. Many studies have demonstrated its potential application in biomedicine for disease diagnosis, cell biology, and therapeutic response monitoring [13,14,15]. Specifically in relation with oral cancers, FTIR imaging has been applied extensively for studying disease progression and diagnostic differentiation [16,17,18]. We were able to identify the changes in collagen fiber networks in the tumor microenvironment (TME) in vitro caused by melanoma and oral squamous carcinoma cells (OSCC) using FTIR imaging [18]. Results suggest that the features present in the amide and collagen triplet regions could serve as a spectral marker for cancer-induced modifications in TME. Benard et al. observed similar results in breast cancers [18,19]. A recent study showed that FTIR imaging could be used for identifying alteration in the protein content and glycosaminoglycan structure in cartilage tissue after irradiation [20].

Irradiation-induced changes in oral mucosa can happen at both morphological and biochemical levels that can lead to acute and chronic adverse effects. An accurate identification of these changes is mandatory for designing appropriate therapeutic interventions. Architectural changes can be identified by studying the microstructure of epithelium. This proof-of-principle study was undertaken to assess the changes in the biochemical milieu of the oral mucosa due to irradiation using FTIR imaging. FTIR measurements in micrometer scale may provide a detailed fingerprint of the biochemical content of the irradiated oral mucosa in a label-free and objective manner.

## 2. Materials and Methods

### 2.1. Clinical Samples

The present study fulfilled the World Medical Association Declaration of Helsinki (Helsinki, Finland, 1964). The ethical committee of the VU University Medical Center in Amsterdam (2011/220) and the Research Ethics Committee of the Northern Savo Hospital District (754/2018) approved the study. All patients signed an informed consent to take part in the study.

The first group “healthy control” (Group 1) consisted of 11 fully edentulous patients with no history of head and neck cancer or radiotherapy. The oncology group consisted of two subgroups. The first sub-group, to referred as “non-irradiated control” (Group 2), consisted of tissue specimens from subjects who had received a surgery for cancer treatment, but were not irradiated.

The second subgroup “irradiated” (Group 3) consisted of biopsies from subjects with surgically removed head and neck cancer and post-operative irradiation therapy or primary irradiation therapy. Patient treatment plans of these subjects were designed individually by the oncological surgery team based on the patient specific parameters. Approximately 1.55–2.0 Gy doses of radiotherapy per session for around 20 sessions were given to each patient as a post-operative irradiation therapy or the maximum dose as a primary irradiation therapy treatment. Total irradiation therapy doses varied between from 40 Gy to 70 Gy. Patients, who received 50 Gy dose or more, also participated in hyperbaric oxygen therapy. Radiation doses were based on the post-operative cone beam-CT image or estimated by the radiotherapist based on the treatment plans. All patients in the oncology groups were fully edentulous who underwent dental rehabilitation with dental implants in the mandible. All irradiated patients received dental implants and had biopsies taken at the irradiated sites. Patients with radiation fields that did not include the mandible and patients who had undergone mandibular reconstruction with osteomyocutaneous grafts were excluded. In the control group, the biopsies were obtained during vestibuloplasty, from the anterior side of the anterior alveolar ridge. Additional and demographic details of patient groups are provided in Table 1.

### 2.2. Light Microscopy (LM)

Tissue samples were gently rinsed with saline solution followed by fixing in 10% phosphate-buffered formalin and embedding in paraffin blocks (FFPE). Staining procedures included deparaffinising, clearing in xylene, and washing by descending alcohol series. The sections were stained with Mayer’s hematoxylin for 15 min followed by counterstaining with eosin for 1 min for clearing and differentiation of the hematoxylin and eosin (HE) staining. Slides were washed, dehydrated by ascending alcohol series, cleared in xylene, and mounted to complete the histology procedure.

### 2.3. Fourier-Transform Infrared (FTIR) Scanning

The specimens were cut into three 4 µm thick slices, then deparaffinized with xylene for 10 min. The HE stained sample was used to pre-select a region of interest (ROI) for spectral analysis. Dried samples were used for FTIR scanning with Spectrum Spotlight 300 Imaging spectrometer (Perkin Elmer, Wellesley, MA, USA) in imaging mode. The samples were scanned from 4000 cm^−1^ to 720 cm^−1^ with 16 scans per pixel and 6.25 µm^2^ pixel size. All the samples were analyzed in the same environmental conditions with 0% relative humidity maintained by a continuous flow of nitrogen in to the measurement chamber. Substrate interference was removed by subtracting the background spectra, which was measured under similar conditions (16 scans), through a portion of the sample containing no material other than the barium fluoride substrate.

### 2.4. Data Analysis

The chemical maps were created with Cytospec version 2.00.03. Spectral pre-processing and principal component analysis (PCA) were performed with in-house developed MATLAB based tools. Baseline corrections, by fitting a polynomial function of 5th order followed by unit normalization, were performed on the spectra extracted from superficial region of tissue. Difference spectra were generated to investigate minor spectral differences.

## 3. Results

### 3.1. Histological Analysis using Light Microscopy

**Figure 1:**
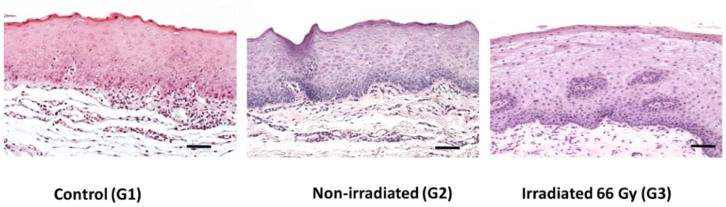
Hematoxylin and Eosin stained images showing differences in the superficial region of control, non-irradiated, and irradiated (Bar = 100 µm).

To understand the changes induced by irradiation, we compared the control and oncology groups. The histological analysis of the healthy control group revealed normal stratified composition along with keratinization of the epithelial layers. There were no signs of inflammation, atypical, or dysplastic cells. Similar microstructural arrangement was observed in “non-irradiated control” samples. In irradiated specimens, the epithelial cell layers appeared thicker, also some of the cells had expanded cytosol and cell nuclei. Typical histological findings of the oral mucosa in different study groups are shown in Figure 1.

### 3.2. FTIR analysis

**Figure 2:**
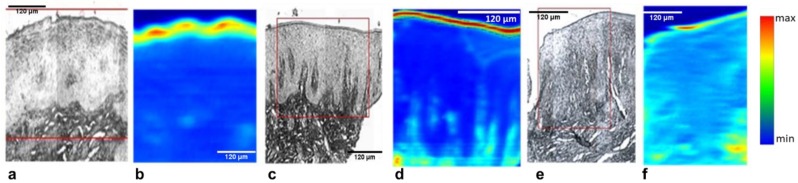
Representative white light (**a**,**c**,**e**) and corresponding FTIR chemical maps (**b**,**d**,**f**) using glycoprotein band (1385 cm^−1^). Areas of spectral extraction in the superficial layer of control (**a**,**b**), non-irradiated (**c**,**d**), and irradiated (**e**,**f**) samples are marked.

The superficial layer is most susceptible to both structural and biochemical damages induced by irradiation. Chemical maps showing distribution of glycoproteins (1385 cm^−1^) across the whole tissue section are shown in Figure 2. As can be seen, irradiated specimens have reduced amount of glycoprotein in the superficial layer, with respect to healthy and non-irradiated controls.

**Figure 3:**
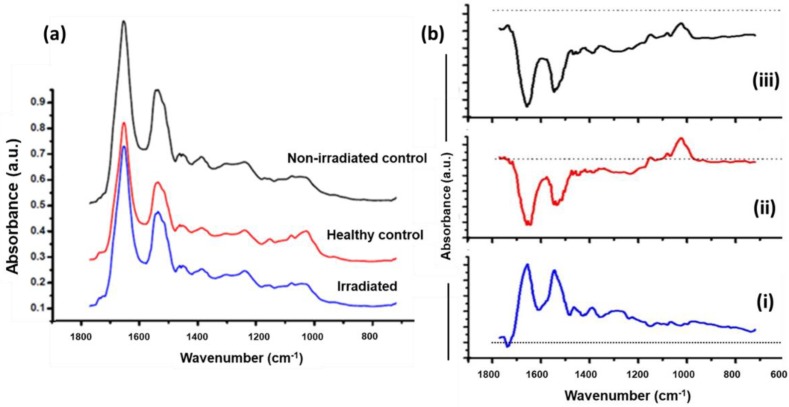
(**a**) Normalized mean spectra extracted from superficial region. Spectra are vertically offset for better visibility. (**b**) Difference spectrum. (i) Non irradiated control–irradiated. (ii) Healthy control–irradiated. (iii) Healthy control–non irradiated control. Dotted line indicates zero axis.

In the next step, average FTIR spectra in the fingerprint range (720–1770 cm^−1^) were extracted from the superficial layers. As shown in Figure 3a, differences in the spectral bands related mostly to nucleic acids, lipids, and proteins were observed. Spectra from healthy control show strong nucleic acid features originating from symmetric and asymmetric stretching of phosphodiester bonds (1030 cm^−1^,1242 cm^−1^) and phosphate (1082 cm^−1^) with respect to irradiated and non-irradiated controls [13,21]. Spectral differences in terms of broadening of amide III (1284 cm^−1^), amide II (1550 cm^−1^), and amide I (1640 cm^−1^) in non-irradiated control and irradiated specimens indicates changes in the protein structural arrangement. Furthermore, to bring out spectral changes among three groups clearly, difference spectra were computed and shown in Figure 3b. This is one of the conventional ways of representing spectral differences that can occur over a selected spectral range and can help in understanding the moieties that are being modified. The subtracted irradiated spectrum (i) has spectral bands belonging to amide I and amide II, suggesting its predominance in non-irradiated controls. Small dips around 1736 cm^−1^ and 1162 cm^−1^ suggests its presence in irradiated specimens. Less nucleic acids and broad amide bands were noted on comparison of irradiated specimens with healthy controls (ii). We have also compared two different controls used in the study (iii). The difference spectrum has positive predominating nucleic acid and lipid features from healthy controls, and negative amide I and amide II bands from non-irradiated control.

**Figure 4:**
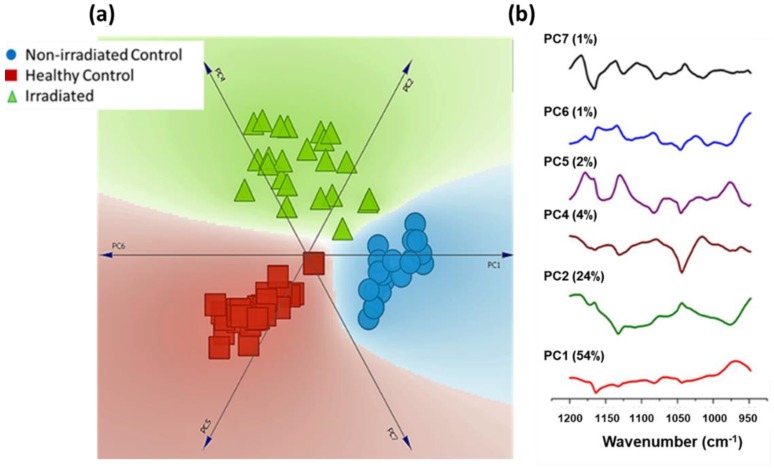
(**a**) Linear projection plot of principal components used for spectral discrimination. (**b**) Loading plot of principal component used for PCA. Variance contribution of each principal component is shown in the parenthesis.

These spectral differences were further utilized to test the feasibility of classification among three groups using principal component analysis (PCA). A total of 39 healthy control, 21 non-irradiated control, and 24 irradiated spectra in the range 950–1200 cm^−1^ were used as input for PCA. As shown in the linear projection plot, exclusive clusters belonging to each patient group were obtained (Figure 4). Loading plots indicating spectral features utilized for classification are also shown. Corroborating with spectral differences from the mean spectra loading plots also indicates nucleic acid (1082 cm^−1^) as the major discriminatory feature. The slight spread of data points in the irradiated group can be attributed to differences in the radiation dose received by each subject.

## 4. Discussion

In tumor cells, RT causes irreversible damages to DNA, lipids, and proteins with the help of free radicals. Following irradiation, there is an increase in the bacterial growth and accumulation of immune cells in the epithelium due to the initiation of the healing process. In our previous studies, we have been able to show the ruptures of cell-cell junctions and imbalances of extracellular links between epithelial cells of oral mucosa after irradiation using scanning and transmission electron microcopy [7,8,9,10]. Our findings suggests that irradiation changes the expression of transmembrane mucin 1 (MUC 1) glycoprotein in the oral epithelium, thereby possibly causing the breakage of the cell-cell junction and destroying the protectable complex of salivary pellicle. Our recent TEM measurements of irradiated oral mucosa suggest presence of enlarged intercellular spaces between oral epithelial cells and unusual fragmented expression of MUC 1. Therefore, we can hypothesize that even though clinical appearance is normal, changes at the biochemical level and structural components are distinct. Irradiation affects the homeostasis and function of protective barrier of oral mucosa.

The present study is aimed towards identifying biochemical differences induced by irradiation in the oral mucosa. We have used light microscopy and FTIR spectroscopy to investigate irradiated human oral mucosa. FTIR spectroscopic techniques have been extensively used to diagnose and differentiate the chemical differences between diseased and healthy tissues based on the IR absorption via the molecular vibrations of biosamples [13,14,15,16,17,18,19,20,21]. Each biomolecule, in principle, has its own distinct pattern of absorption peaks, which can be used as a fingerprint for molecular identification. Furthermore, the intensities of the absorption peaks are directly proportional to the concentration of components in a mixture; thus, IR spectroscopy serves both as a qualitative and as a quantitative spectroscopic tool. Biochemical changes identified by FTIR imaging could add a significant value to the histopathological assessment of oral epithelium in the superficial layer in response to irradiation. As the technique does not involve the use of any labels, it can be readily translated for routine clinical applications. The differences observed in glycoprotein chemical maps across the tissue section further corroborates with the already mentioned structural deformities in MPL induced by irradiation. Spectral difference observed in the present study suggests changes in nucleic acid and protein can be considered as an indirect marker for irradiation damage in mucosa. Further, we have also observed differences among ‘non-irradiated control’ and ‘healthy control’ specimens. This could be an indication of the earliest neoplastic transformations in the mucosa due to ‘field cancerization’ [22,23,24]. Previous studies on oral cancer subjects have demonstrated that uninvolved mucosa of cancer subjects could harbor minor, invisible changes that could make the mucosa biochemically different from healthy controls [20]. Differences in nucleic acid and protein observed among ‘non-irradiated control’ and ‘healthy control’ specimens could be an indication of increased inflammation and early DNA damage [21,22].

One of the main benefits of employing label-free methods for clinical applications is the objectivity. The spectral differences among three groups were further utilized for assessing feasibility of classification using Principal Component Analysis (PCA). A number of machine learning algorithms, such as linear discriminant analysis, support vector machine, and partial least square analysis, have been employed for the discrimination of the spectral data. Principal component analysis (PCA) is a routinely employed mathematical approach for data compression and visualization. It describes data variance by identifying a new set of orthogonal features, called principal components (PCs) or factors. The first PC is chosen in the direction where maximum variability in the data can be explained. The subsequent orthogonal PCs are chosen on the basis of their contribution to the remaining variability. For visual discrimination, we project each of the spectra in the newly formed co-ordinate space of selected PCs [25]. As shown in Figure 4, clear discrimination between three groups were observed in the linear projection plot. This visualization method maps the PC data dimensions onto a two dimensional space for the purpose of clustering. The PC scores describing specimen characteristics are placed around the perimeter of the circle, which also provide dimension anchors [26,27]. Loading are essentially the combination of directions and weights of each PCs. A loading plot can provide information about biochemical components responsible for classification in a particular direction or factor. Consistent with previously mentioned differences in the spectral profiles of “healthy control”, ”non-irradiated control”, and “irradiated” changes in nucleic acid and amide features appear in the major discriminatory features in these PCs. Our current efforts are inclined towards analyzing chronic variants of irradiation damage, e.g., mandibular osteoradionecrosis.

Overall, findings of this preliminary study suggest that FITR imaging could be useful, non-disruptive, and a novel technique to measure biochemical changes, assess the state of oral mucosa in different irradiation induced complications, and the suitability of oral mucosa before implantation.

## Figures and Tables

**Table 1:** Demographic data of 25 subjects.

Patients	Number (M/F)	Age (Mean; SD)
Healthy control	11 (7/4)	33–74 (59.9; 11.2) years
Non-Irradiated control	5 (4/1)	57–80 (65.8; 8.6) years
Irradiated	9 (5/4)	58–82 (70.4; 8.2) years
